# High-cost users still came to hospitals during the COVID-19 pandemic during first wave data in Thailand: secondary data analysis

**DOI:** 10.1186/s12889-024-20325-y

**Published:** 2024-10-22

**Authors:** Picharee Karunayawong, Piyada Gaewkhiew, Myka Harun Sarajan, Chulathip Boonma, Rukmanee Butchon, Jarawee Sukmanee, Thanayut Saeraneesopon, Yot Teerawattananon, Wanrudee Isaranuwatchai

**Affiliations:** 1 Health Intervention and Technology Assessment Program (HITAP), Ministry of Public Health, Nonthaburi, Thailand; 2 Department of Community Dentistry, Faculty of Dentistry, Mahidol University, Bangkok, Thailand; 3 Saw Swee Hock School of Public Health, National University of Singapore, 12 Science Drive 2, #10-01, Singapore, 117549 Singapore; 4 Institute of Health Policy, Management and Evaluation, University of Toronto, Toronto, Canada

**Keywords:** COVID-19, Big Data, High-Cost Users, Thailand, Health Expenditure, Data Science

## Abstract

**Background:**

The phenomenon of high-cost users (HCUs) in health care occurs when a small proportion of patients account for a large proportion of health care expenditures. By understanding this phenomenon during the COVID-19 pandemic, tailored interventions can be provided to ensure that patients receive the care they need and reduce the burden on the health system.

**Objectives:**

This study aimed to determine (1) whether the HCUs phenomenon occurred during the pandemic in Thailand by exploring the pattern of inpatient health expenditures over time from 2016 to 2021; (2) the patient characteristics of HCUs; (3) the top 5 primary diagnoses of HCUs; and (4) the potential predictors associated with being an HCU.

**Methods:**

The secondary data analysis was conducted via inpatient department (IPD) e-Claim data from the National Health Security Office for the Universal Coverage Scheme, which provides health care to ~ 80% of the Thai population. Health care expenditure over time was calculated, and the characteristics of the population were examined via descriptive analysis. Multinomial logistic regression was applied to explore the potential predictors associated with being an HCU.

**Results:**

The characteristics of HCUs remained relatively the same from 2016 to 2021. In terms of the proportion of male (55%) to female patients (45%), the age ranged from 55 to 57 years, with an estimated 8-day length of hospital stay and 7 admissions per year, and the average health care cost per patient was ≥ USD 2,860 (100,000 THB). The low-cost users (LCUs) group (the bottom 50% of the population), had more female patients (55%), a younger age ranging from 27 to 33 years, a 3-day length of stay, 1‒2 admissions per year, and a lower average health care cost per patient, which was less than USD 315 (≤ 11,000 THB).

**Conclusion:**

The HCUs phenomenon still existed even with limited health care accessibility or lockdown measures implemented during the COVID-19 pandemic. This finding could indicate the uniqueness of the need for health services by HCUs, which differ from those of other population groups. By understanding the trends of health care utilization and expenditure, along with potential predictors associated with being an HCU, policies can be introduced to ensure the appropriate allocation of health resources to the right people in need of the right care during future pandemics.

**Supplementary Information:**

The online version contains supplementary material available at 10.1186/s12889-024-20325-y.

## Background

In the landscape of the health care system, health care spending per person each year could reflect the consumption of resources for each treatment or health service, and health care spending appears to be increasing gradually each year. The “high-cost users (HCUs)” phenomenon occurs when a small proportion of the country’s population or approximately 5% of the total population accounts for a large proportion of the national health care expenditure [1]. In a previous study in the USA [1], HCUs used 55% of health care expenditures for various reasons, such as being readmitted to hospitals several times for the same health condition. Furthermore, HCUs appear to have comorbidities that require complex (and potentially more expensive) interventions [2]. These observations were found not only in Western countries but also in Asia, including Singapore, China, and Thailand [3, 4]. Previous research has supported the importance of identifying HCUs to prevent and provide tailored case management for this population [1].

Previous work in Thailand confirmed the HCUs phenomenon, in which 5% of patients accounted for half of health care expenditures in Thailand [5]. The study findings call attention to potential initiatives that can help monitor the magnitude and trends of high-cost use and develop policies to prevent high-cost use. By understanding the HCUs phenomenon, tailored interventions as well as preventive services can be provided to ensure that these patients receive early and proper care. There is a need to mitigate high-cost consequences and thus reduce the burden imposed on the health care system.

After the COVID-19 pandemic started in 2020, there were significant alterations in the use of health care services in Thailand following the implementation of measures such as lockdowns, including deferring nonurgent care, avoiding inpatient admission, and cancelling elective surgery [6–8]. Moreover, it was estimated that during the pandemic, the total global expenditures in the health care system, including low- and middle-income countries (LMICs), ranged from US$130–231 billion to US$0.6–1 trillion annually, depending on different scenarios. [9]. During the pandemic, health professionals continued to provide health care services to the population [10, 11]. The understanding of HCUs’ characteristics could help in understanding patient profiles and health care needs, which is the fundamental element of intervention and policy in health care [12]. In addition, tailored interventions for HCUs could reduce the use of scarce resources and meet unmet clinical needs [1].

To support efforts toward an efficient health care system, evidence of the high-cost user phenomenon in Thailand is needed to increase health care policy-makers and system efficiency. This study aimed to study the phenomenon of HCUs during the COVID-19 pandemic in an upper-middle income country (Thailand) by exploring the patterns of inpatient health expenditures over time from 2016 to 2021. Second, this study aimed to explore the patient characteristics of HCUs, including demographic data, residential regions, the top five primary diagnoses of HCUs, and the potential predictors associated with being an HCU in a hospitalized population.

## Methods

### Data sources

A de-identified and anonymized hospital reimbursement database at the National Health Security Office (NHSO) was accessed by the research team for the study. This study used data on hospital admissions, disease diagnoses, length of hospital stay, and total medical expenditures, which consists of all inpatient cases (covered under the UCS) from January 2016 to October 2021. The NHSO provides public health insurance under the Universal Coverage Scheme (UCS), which covers approximately 80% of the Thai population [13].

### Case definition

The years when patients were discharged were used for analysis (Supplementary file 1). The cost per patient was evaluated over time, from January 2016 to the year of discharge (Supplementary file 2). The cost per patient, including the total cost per admission paid by the NHSO, was then calculated, and patients were grouped into 100 sections as percentiles. The proportions for all patients and these individuals were categorized into the following percentiles according to a previous study in which the top 5% of the total population was categorized as HCUs [5, 14–16]. All patients were grouped as follows: (1) high-cost users (HCUs) between the 96th and 100th percentiles (top 5% of the population); (2) average-cost users (ACUs): between the 51st and 95th percentiles; and (3) low-cost users (LCUs): from the 1st to the 50th percentiles (bottom 50% of the population).

### Statistical analysis

Descriptive analyses were conducted to analyze hospitalization costs and patient characteristics for HCUs, ACUs, and LCUs and to identify the top diagnoses for each group. A multinomial logistic regression model was used to investigate the potential predictors associated with being an HCU and ACU compared with an LCU. A p-value < 0.05 was used to indicate statistical significance. All analyses were conducted via Stata (Release 16, College Station, Texas, USA) [14] and R (Vienna, Austria) [17].

## Results

### Patient characteristics

A secondary data analysis of 19,967,341 patients (from 30,452,449 hospitalization records) over a 6-year period from January 2016 to October 2021 was performed. The patient characteristics of the HCUs, ACUs, and LCUs were compared in two periods: (1) the prepandemic period (2016 to 2019) and (2) the pandemic period (2020 to 2021) (Table 1). The demographic profiles of HCUs, ACUs, and LCUs in the pandemic period did not vary from the prepandemic period in terms of the proportion of male to female patients (~ 55% to ~ 45% in the HCU group, ~ 50% to ~ 50% in the ACU group, and ~ 45% to ~ 55% in the LCU group); age (~ 56 years in the HCU group, ~ 47 in the ACU group, and ~ 26 years in the LCU group); average length of stay per admission (~ 8 days in the HCU group, ~ 5 days in the ACU group, and ~ 3 days in the LCU group); average number of admissions per patient (~ 7 admissions in the HCU group, ~ 3 admissions in the ACU group, and ~ 1 admission in the LCU group); and average cost in Thai Baht (THB) per patient (≥ 100,000 THB in the HCU group and ~ 3,000 THB in the LCU group, where the average exchange rate was 31.99 THB per USD in 2021). The average cost of ACUs increased from ~ 16,000 THB (2016 to 2019) to ~ 19,000 THB in 2020 and ~ 32,000 THB in 2021.

**Table 1:** Demographic profile of High-Cost Users (HCUs), Average-Cost Users (ACUs), and Low-Cost Users (LCUs) from 2016 to 2019 (pre-pandemic period) and 2020 to 2021 (pandemic period)

**Variables**	**2016**			**2017**			**2018**		
	**HCUs**	**ACUs**	**LCUs**	**HCUs**	**ACUs**	**LCUs**	**HCUs**	**ACUs**	**LCUs**
**Number of** **male to female (%)**	94,636 (54.85%): 77,893 (45.15%)	743,725 (47.90%): 809,061 (52.10%)	773,799 (44.85%): 951,516 (55.15%)	93,270 (55.04%): 76,188 (44.96%)	737,181 (48.33%): 788,101 (51.67%)	760,593 (44.88%): 934,147 (55.12%)	96,560 (54.98%): 79,069 (45.02%)	771,178 (48.79%): 809,483 (51.21%)	798,069 (45.44%): 958,222 (54.56%)
**Age (years), mean ± SD** **(min–max)**	55.10 ± 21.70 (0 – 107)	46.08 ± 26.56 (0 – 117)	23.62 ± 25.75 (0 – 115)	55.52 ± 21.49 (0 – 118)	46.72 ± 26.33 (0–117)	24.97 ± 26.24 (0 – 111)	55.98 ± 21.36 (0 – 117)	46.44 ± 26.74 (0 – 119)	24.73 ± 26.14 (0 – 117)
**Average length** **of stay (days)** **per admission, mean** ** ± SD (min–max)**	7.98 ± 14.00 (0 – 589)	4.29 ± 6.73 (0 – 577)	2.53 ± 2.17 (0 – 589)	7.87 ± 14.08 (0 – 959)	4.41 ± 7.40 (0 – 797)	2.55 ± 2.20 (0 – 507)	7.66 ± 13.69 (0 – 1173)	4.35 ± 7.33 (0 – 1112)	2.54 ± 2.29 (0 – 857)
**Average no. of admissions per patient, mean ± SD** **(min–max)**	6.50 ± 5.85 (1 – 104)	2.48 ± 1.95 (1 – 25)	1.10 ± 0.30 (1 – 5)	6.77 ± 6.05 (1–110)	2.53 ± 2.01 (1 – 25)	1.10 ± 0.31 (1 – 5)	6.82 ± 6.14 (1 – 107)	2.58 ± 2.05 (1 – 24)	1.10 ± 0.31 (1 – 6)
**Average cost in THB per patient, mean ± SD** **(min–max)**	117,737 ± 76,328 (57,086 – 1,995,684)	14,876 ± 11,213 (4,865 – 57,086)	2,627 ± 1,014 (0.07 – 4,865)	117,737 ± 76,328 (57,086 – 1,995,684)	14,876 ± 11,213 (4,865 – 57,086)	2,627 ± 1014 (0.07 – 4,865)	122,321 ± 77,441 (61,241 – 3,249,873)	16,142 ± 12,226 (5,397 – 61,241)	2,912 ± 1,123 (0.8 – 5,397)
**Hospital type**									
**Provincial hospital: Tertiary care (Center)**^**a**^	194,554 (31.31%)	625,862 (24.01%)	279,707 (15.44%)	198,908 (31.20%)	620,411 (23.90%)	286,569 (16.10%)	208,290 (31.44%)	638,545 (23.43%)	289,318 (15.69%)
**Provincial/District hospital (General)**^**b**^	116,741 (18.79%)	654,888 (25.12%)	386,397 (21.33%)	125,847 (19.74%)	6574,33 (25.32%)	387,956 (21.80%)	133,479 (20.15%)	692,047 (25.39%)	394,315 (21.38%)
**Community**^**c**^	172,122 (27.70%)	1,023,239 (39.25%)	996,445 (55.01%)	179,654 (28.18%)	1,044,984 (40.25%)	955,627 (53.70%)	193,213 (29.17%)	1,119,835 (41.08%)	1,008,482 (54.69%)
**Private/ Clinic**	30,409 (4.89%)	91,594 (3.51%)	48,166 (2.66%)	26,075 (4.09%)	73,533 (2.83%)	47,369 (2.66%)	23,367 (3.53%)	68,413 (2.51%)	46,995 (2.55%)
**Outside MOPH**	107,420 (17.29%)	204,564 (7.85%)	99,071 (5.47%)	107,064 (16.79%)	198,704 (7.65%)	100,774 (5.66%)	104,043 (15.71%)	205,814 (7.55%)	103,157 (5.59%)
**Other**	198 (0.03%)	7,034 (0.27%)	1,453 (0.08%)	74 (0.01%)	978 (0.04%)	1,334 (0.07%)	42 (0.01%)	1,114 (0.04%)	1,693 (0.09%)
**Variables**	**2019**			**Pandemic period of 2020**			**Pandemic period of 2021**		
	**HCUs**	**ACUs**	**LCUs**	**HCUs**	**ACUs**	**LCUs**	**HCUs**	**ACUs**	**LCUs**
**Number of male to female (%)**	97,714 (54.90%): 80,270 (45.10%)	786,509 (49.10%): 815,362 (50.90%)	813,680 (45.72%): 966,175 (54.28%)	87,484 (55.20%): 71,011 (44.80%)	710,297 (49.80%): 716,107 (50.20%)	711,565 (44.89%): 873,441 (55.11%)	85,663 (52.51%): 77,470 (47.49%)	737,295 (50.22%): 730,921 (49.78%)	753,846 (46.21%): 877,507 (53.79%)
**Age (years), mean ± SD (min–max)**	56.30 ± 20.98 (0 – 112)	47.84 ± 26.23 (0 – 117)	25.46 ± 26.25 (0 – 115)	57.00 ± 20.00 (0 – 115)	50.26 ± 24.76 (0 – 116)	27.64 ± 26.98 (0 – 118)	54.52 ± 20.95 (0 – 109)	47.76 ± 24.12 (0 – 117)	33.15 ± 27.83 (0 – 122)
**Average length of stay (days) per admission, mean ± SD (min–max)**	7.56 ± 13.81 (0 – 1360)	4.31 ± 7.29 (0 – 1653)	2.53 ± 2.20 (0 – 366)	7.37 ± 13.30 (0 – 967)	4.48 ± 7.30 (0 – 1041)	2.54 ± 2.30 (0 – 511)	8.68 ± 13.72 (0—682)	6.37 ± 7.77 (0 – 1462)	2.93 ± 3.55 (0 – 834)
**Average no. of admissions per patient, mean ± SD (min–max)**	6.84 ± 6.08 (1 – 105)	2.60 ± 2.10 (1 – 25)	1.10 ± 0.31 (1 – 5)	7.00 ± 6.46 (1 – 98)	2.62 ± 2.18 (1 – 25)	1.11 ± 0.33 (1 – 4)	6.50 ± 7.19 (1 – 125)	2.63 ± 2.38 (1 – 34)	1.17 ± 0.43 (1 – 8)
**Average cost in THB per patient, mean ± SD (min–max)**	125,061 ± 76,182 (63,632 – 2,144,568)	16,815 ± 12,800 (5,550 – 63,632)	2,982 ± 1,145 (0.01 – 5,550)	139,985 ± 80,924 (73,466 – 3,074,660)	19,554 ± 15,001 (6,430 – 73,466)	3,276 ± 1,357 (0.57 – 6,430)	188,997 ± 103,715 (105,749 – 5,260,359)	32,603 ± 22,158 (10,571 – 105,748)	4,765 ± 2,614 (3 – 10,571)
**Hospital type**									
**Provincial hospital: Tertiary care (Center)**^**a**^	211,637 (31.61%)	645,561 (23.36%)	285,830 (15.29%)	201,184 (33.00%)	588,724 (24.08%)	258,681 (15.43%)	152,487 (31.41%)	584,987 (24.18%)	303,152 (17.17%)
**Provincial/District hospital (General)**^**b**^	134,040 (20.02%)	700,046 (25.33%)	396,523 (21.21%)	122,986 (20.17%)	635,044 (25.98%)	360,915 (21.52%)	100,482 (20.70%)	593,494 (24.53%)	410,721 (23.26%)
**Community**^**c**^	194,999 (29.12%)	1,141,344 (41.29%)	1,042,456 (55.75%)	167,049 (27.40%)	975,695 (39.91%)	929,830 (55.45%)	128,365 (26.44%)	971,888 (40.17%)	917,025 (51.93%)
**Private/ Clinic**	24,267 (3.62%)	65,763 (2.38%)	44,807 (2.40%)	22,892 (3.75%)	53,265 (2.18%)	33,203 (1.98%)	22,566 (4.65%)	47,573 (1.97%)	25,764 (1.46%)
**Outside MOPH**	104,594 (15.62%)	210,219 (7.61%)	98,102 (5.25%)	95,521 (15.67%)	191,239 (7.82%)	93,191 (5.56%)	81,471 (16.78%)	216,653 (8.95%)	107,625 (6.10%)
**Other**	37 (0.01%)	1,042 (0.04%)	2,084 (0.11%)	63 (0.01%)	821 (0.03%)	1,127 (0.07%)	53 (0.01%)	4,766 (0.20%)	1,503 (0.09%)

*HCUs* High-Cost Users (the 96th -100th percentile), *ACUs* Average-Cost Users (the 51st -95th percentile), *LCUs* Low-Cost Users (the 1st -50th percentile)

^a^capacity: more than 500 beds

^b^capacity: 200–500 beds

^c^capacity: 10–150 beds

**Fig. 1:**
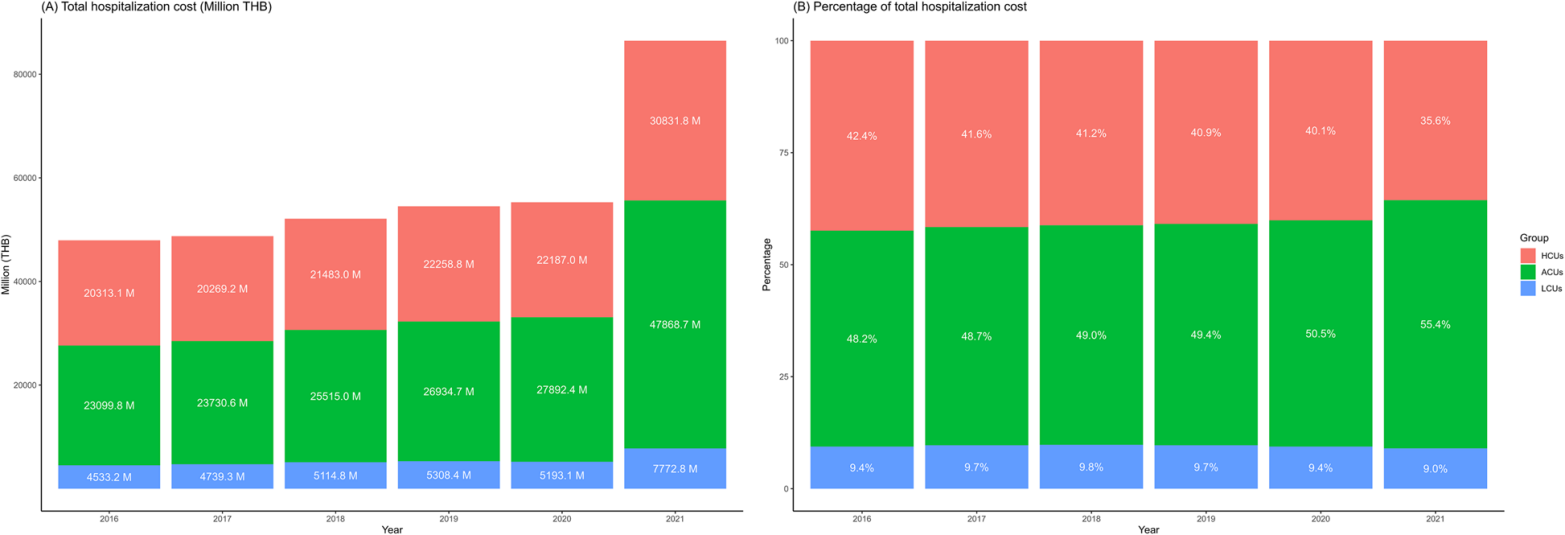
**A** Total hospitalization cost (million THB) of inpatient patients from 2016 to 2021 and (**B**) Percentage of total hospitalization cost from 2016 to 2021. *Notes*: HCUs: High-Cost Users (the 96th -100th percentile); ACUs: Average-Cost Users (the 51st -95th percentile); LCUs: Low-Cost Users (the 1st -50th percentile); average rate in 2021: 31.99 THB per USD

Thailand experienced an increasing trend in hospitalization expenditures, especially the rapid increase during the COVID-19 pandemic in 2021 (Fig. 1A). Figure 1B shows that the top 5% of the hospitalized population (HCUs within and above the 96th percentile of all hospitalized patients) utilized close to 40% of the annual health expenditure, confirming that the HCUs phenomenon still existed even during the pandemic year (2020–2021). On the other hand, the average-cost users (ACUs or individuals between the 51st and 95th percentiles) accounted for approximately 50% of the annual hospitalization expenditure. Individuals within the 50th percentile or low-cost users (LCUs) accounted for only approximately 10% of the hospitalization expenditure.

**Fig. 2:**
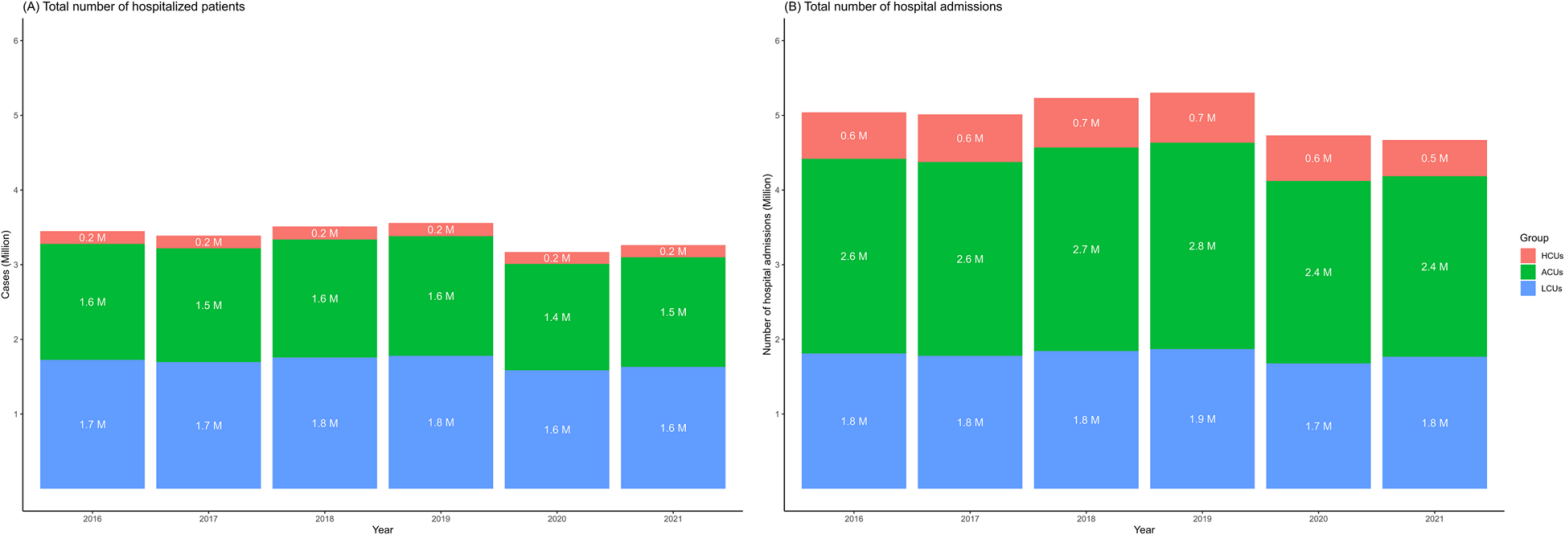
**A** Total number of hospitalized patients from 2016 to 2021 and (**B**) Total number of hospital admissions from 2016 to 2021. *Notes*: HCUs: High-Cost Users (the 96th -100th percentile); ACUs: Average-Cost Users (the 51st -95th percentile); LCUs: Low-Cost Users (the 1st -50.^th^ percentile)

Figure 2A shows the total number of patients admitted to the hospital during 2016 and 2021, highlighting the slightly increasing trend in the prepandemic period before a slight reduction in hospitalization between 2020 and 2021, supporting the common observation of a reduction in hospitalization during the COVID-19 pandemic. A reduction in admissions during the pandemic was associated with increased total hospitalization costs (as shown previously). Furthermore, Fig. 2B shows the total number of hospital admissions of patients, where one patient could have more than one admission, which is reflected via the total number of hospital admissions for ACUs and HCUs (green and orange areas) who were admitted to a hospital being greater than that for LCUs (blue area) [ACUs + HCUs > LCUs].

### Top primary diagnoses for HCUs, ACUs, and LCUs

**Fig. 3:**
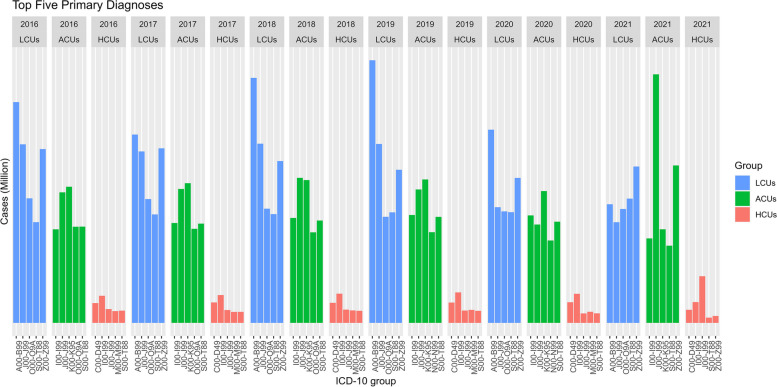
Top five primary diagnoses between 2016 to 2021. Notes: HCUs: High-Cost Users (the 96th -100th percentile); ACUs: Average-Cost Users (the 51st -95th percentile); LCUs: Low-Cost Users (the 1st -50.^th^ percentile); ICD10 code described as diseases (Supplementary 3)

The top five primary diagnoses (identified on the basis of ICD-10 codes) across HCUs, ACUs, and LCUs did not change over time from 2016 to 2020; only the change in order within each group occurred. The most common diagnosis for HCUs was diseases of the circulatory system, followed by neoplasms, from 2016 to 2020. For LCUs, certain infectious and parasitic diseases were the most common diagnoses from 2016 to 2020. For ACUs, diseases of the digestive system were the most common diagnoses from 2016 to 2020, except in 2018. During the pandemic year (2021), there was a change in the most common diagnoses in all groups—respiratory-related diseases in the HCU and ACU groups and other conditions that influence health status in the LCU group. Moreover, there was a change in the top five diagnoses of ACUs, which was the substitution of diseases of the genitourinary system for pregnancy, childbirth, and puerperium, in 2019 and 2020 (Fig. 3 and Supplementary file 3).

### Potential predictors of being an HCU, ACU, and LCU

Table 2 presents the potential predictors of being an HCU and ACU compared with an LCU from 2016–2021. HCUs tended to be older, and they had a greater risk of mortality and a greater number of comorbidities, as measured by the Charlson comorbidity score. Additionally, HCUs were more frequently admitted to hospitals outside the Ministry of Public Health (MoPH). In terms of primary diagnosis, HCUs had a higher incidence of respiratory or circulatory diseases, neoplasms, injuries, or muscular diseases as their primary diagnosis (except in 2021). HCUs had a greater annual number of admissions and were more likely to die in hospitals than LCUs. With respect to hospital admissions, during the period from 2018 to 2021, HCUs were admitted primarily to hospitals in the central region rather than in Bangkok and metropolitan areas. However, in 2021, compared to LCUs, HCUs were admitted to private hospitals or clinics more than central hospitals.

**Table 2:** Potential predictors of HCUs, ACUs, and LCUs using multinomial logistic regression

**Users group by cost levels**	**Cohort**	**2016**			**2017**			**2018**				
	**N**	5,039,864			5,013,294			5,232,162				
	R^**2**^	0.44			0.4381			0.4413				
		**RRR**	**[95% conf. interval]**		**RRR**	**[95% conf. interval]**		**RRR**	**[95% conf. interval]**			
**LCUs**		(base)			(base)			(base)				
**ACUs**												
**Age**		1.029*	1.029	1.029	1.027*	1.027	1.027	1.028*	1.028			1.028
**Gender (vs Male)**												
	Female	0.816*	0.811	0.820	0.801*	0.796	0.805	0.810*	0.806			0.815
**Hospital type** **(vs Center Hospital**^**a**^**)**												
	General^b^	0.721*	0.716	0.727	0.763*	0.757	0.769	0.785*	0.779			0.792
	Community^c^	0.224*	0.222	0.226	0.260*	0.258	0.262	0.265*	0.263			0.267
	Private/ Clinic	0.691*	0.680	0.703	0.581*	0.571	0.592	0.547*	0.538			0.557
	Outside MoPH	1.174*	1.159	1.189	1.168*	1.153	1.183	1.218*	1.203			1.234
	Others	10.917*	8.973	13.282	1.097*	0.826	1.455	4.012*	2.687			5.992
**Region (vs BKK and Metropolitan)**												
	Central	0.885*	0.873	0.897	0.987	0.973	1.000	0.995	0.982			1.009
	East	1.005*	0.990	1.020	0.941*	0.927	0.955	0.989	0.975			1.003
	North	1.045*	1.032	1.058	0.987	0.975	1.000	1.108*	1.095			1.122
	North East	1.065*	1.052	1.078	0.987*	0.975	0.999	1.130*	1.117			1.143
	South	0.949*	0.937	0.961	0.865*	0.854	0.876	0.903*	0.891			0.914
	Not Applicable	0.034*	0.028	0.043	0.230*	0.169	0.313	0.056*	0.037			0.085
**Charlson Comorbidity**		1.621*	1.604	1.638	1.629*	1.612	1.645	1.632*	1.616			1.649
**Discharge Status (vs Alive)**												
	Death	1.562*	1.547	1.577	1.667*	1.650	1.684	1.812*	1.792			1.832
**Primary Diagnosis (vs Disease-free)**												
	Respiratory	0.894*	0.887	0.901	0.942*	0.935	0.950	1.045*	1.036			1.053
	Circulatory	1.231*	1.214	1.248	1.362*	1.344	1.380	1.459*	1.440			1.479
	Neoplasms	7.660*	7.404	7.925	7.093*	6.870	7.324	7.005*	6.786			7.232
	Immunity	0.179*	0.177	0.182	0.184*	0.181	0.186	0.209*	0.206			0.212
	Injury	1.098*	1.088	1.108	1.070*	1.060	1.079	1.127*	1.117			1.137
	Muscular	2.279*	2.234	2.325	1.999*	1.962	2.037	1.928*	1.893			1.964
**Number of Visit per year**		13.304*	13.218	13.391	12.463*	12.384	12.542	13.462*	13.377			13.547
**Constant Term**		0.031	0.030	0.031	0.034	0.033	0.034	0.026	0.026			0.026
**HCUs**												
**Age**		1.037*	1.037	1.037	1.035*	1.035	1.035	1.038*	1.037			1.038
**Gender (vs Male)**												
	Female	0.603*	0.598	0.609	0.588*	0.583	0.594	0.597*	0.592			0.602
**Hospital type** **(vs Center Hospital**^a^**)**												
	General^b^	0.441*	0.435	0.446	0.499*	0.493	0.505	0.522*	0.516			0.528
	Community^c^	0.092*	0.091	0.093	0.110*	0.109	0.112	0.116*	0.115			0.118
	Private/ Clinic	0.815*	0.796	0.836	0.693*	0.675	0.711	0.645*	0.628			0.662
	Outside MoPH	2.062*	2.025	2.099	2.236*	2.196	2.277	2.279*	2.239			2.320
	Others	2.491*	1.912	3.247	1.729*	1.086	2.751	1.764*	0.776			4.009
**Region (vs BKK and Metropolitan)**												
	Central	0.770*	0.754	0.786	0.984	0.964	1.005	1.006		0.985		1.026
	East	0.898*	0.878	0.918	0.863*	0.845	0.883	0.917*		0.898		0.938
	North	0.870*	0.854	0.886	0.848*	0.832	0.864	1.044*		1.025		1.064
	North East	0.842*	0.828	0.857	0.801*	0.787	0.815	0.960*		0.943		0.977
	South	0.737*	0.722	0.751	0.699*	0.686	0.713	0.740*		0.725		0.754
	Not Applicable	0.061*	0.041	0.090	0.025*	0.014	0.046	0.028*		0.012		0.069
**Charlson Comorbidity**		2.443*	2.413	2.473	2.492*	2.462	2.521	2.523*		2.494		2.553
**Discharge Status (vs Alive)**												
	Death	3.223*	3.184	3.262	3.373*	3.331	3.415	3.720*		3.671		3.769
**Primary Diagnosis (vs Disease-free)**												
	Respiratory	1.379*	1.361	1.397	1.396*	1.377	1.415	1.558*		1.537		1.578
	Circulatory	3.784*	3.721	3.848	4.151*	4.083	4.220	4.441*		4.369		4.514
	Neoplasms	18.799*	18.141	19.480	18.987*	18.359	19.636	15.996*		15.470		16.540
	Immunity	0.523*	0.506	0.540	0.535*	0.517	0.553	0.610*		0.590		0.631
	Injury	3.503*	3.447	3.559	3.255*	3.204	3.307	3.580*		3.525		3.636
	Muscular	13.814*	13.479	14.158	11.376*	11.108	11.650	12.519*		12.231		12.813
**Number of Visit per year**		19.963*	19.831	20.096	18.721*	18.600	18.843	20.029*		19.900		20.158
**Constant Term**		0.001	0.001	0.001	0.001	0.001	0.001	0.000		0.000		0.001
**Users group by cost levels**	**Cohort**	**2019**			**2020**			**2021**				
	**N**	**5,303,351**			**4,731,430**			**4,670,570**				
	R^**2**^	**0.4417**			**0.434**			**0.3322**				
		**RRR**	**[95% conf. interval]**		**RRR**	**[95%conf. interval]**		**RRR**		**[95%conf. interval]**		
**LCUs**		(base)			(base)			(base)				
**ACUs**												
**Age**		1.029*	1.029	1.029	1.028*	1.028	1.028	1.022*		1.021		1.022
**Gender (vs Male)**												
	Female	0.802*	0.798	0.806	0.750*	0.746	0.754	0.865*		0.860		0.869
**Hospital type** **(vs Center Hospital**^**a**^**)**												
	General^b^	0.786*	0.779	0.792	0.775*	0.768	0.781	0.801*			0.795	0.807
	Community^c^	0.264*	0.262	0.266	0.248*	0.246	0.250	0.409*			0.407	0.412
	Private/ Clinic	0.484*	0.475	0.493	0.522*	0.512	0.533	0.786*			0.771	0.801
	Outside MoPH	1.247*	1.231	1.263	1.131*	1.116	1.146	0.954*			0.943	0.965
	Others	1.291*	0.960	1.736	0.725*	0.534	0.985	1.970*			1.741	2.229
**Region (vs BKKand Metropolitan)**												
	Central	0.971*	0.958	0.984	1.006	0.992	1.020	0.825*			0.815	0.835
	East	0.940*	0.926	0.953	0.996	0.981	1.011	0.968*			0.956	0.981
	North	0.998	0.986	1.011	1.079*	1.065	1.093	0.635*			0.627	0.642
	North East	1.014*	1.002	1.026	1.035*	1.022	1.048	0.645*			0.638	0.652
	South	0.924*	0.912	0.935	0.898*	0.886	0.910	0.810*			0.801	0.819
	N/A	0.116*	0.085	0.158	0.450*	0.329	0.614	0.181*			0.157	0.208
**Charlson Comorbidity**		1.710*	1.693	1.727	1.605*	1.589	1.621	1.150*			1.140	1.160
**Discharge Status (vs Alive)**												
	Death	2.149*	2.122	2.176	3.032*	2.982	3.084	3.496*			3.424	3.570
**Primary Diagnosis (vs Disease-free)**												
	Respiratory	0.945*	0.937	0.952	1.026*	1.016	1.035	7.076*			7.022	7.130
	Circulatory	1.447*	1.428	1.466	1.467*	1.448	1.486	1.409*			1.393	1.424
	Neoplasms	7.020*	6.799	7.247	6.238*	6.053	6.428	4.982*			4.881	5.086
	Immunity	0.211*	0.208	0.214	0.209*	0.206	0.212	4.316*			4.282	4.351
	Injury	1.130*	1.120	1.140	1.111*	1.101	1.121	1.293*			1.282	1.305
	Muscular	2.051*	2.014	2.089	1.942*	1.907	1.978	2.470*			2.427	2.514
**Number of Visit per year**		13.059*	12.978	13.141	9.563*	9.505	9.622	5.581*			5.555	5.608
**Constant Term**		0.027	0.027	0.028	0.039	0.038	0.040	0.053			0.052	0.054
**HCUs**												
**Age**		1.038*	1.039	1.039	1.038*	1.038	1.039	1.029*			1.028	1.029
**Gender (vs Male)**												
	Female	0.590	0.585	0.595	0.552	0.548	0.557	0.738			0.732	0.745
**Hospital type** **(vs Center Hospital**^**a**^**)**												
	General^b^	0.509	0.502	0.515	0.491	0.485	0.498	0.595			0.588	0.602
	Community^c^	0.114	0.113	0.115	0.106	0.105	0.108	0.210			0.208	0.213
	Private/ Clinic	0.586	0.571	0.602	0.764	0.743	0.786	1.724*			1.680	1.769
	Outside MoPH	2.267*	2.227	2.308	2.096*	2.058	2.136	1.462*			1.438	1.487
	Others	0.546	0.271	1.101	0.489	0.218	1.096	0.109*			0.075	0.159
**Region (vs BKK and Metropolitan)**												
	Central	1.021*	1.001	1.042	1.143*	1.119	1.168	0.835*			0.819	0.851
	East	0.875*	0.856	0.894	0.997	0.975	1.020	0.798*			0.782	0.815
	North	0.937*	0.920	0.954	1.123*	1.102	1.145	0.487*			0.479	0.496
	North East	0.847*	0.832	0.862	0.978*	0.960	0.996	0.504*			0.496	0.513
	South	0.827*	0.811	0.843	0.863*	0.846	0.881	0.662*			0.650	0.674
	Not Applicable	0.052*	0.024	0.111	0.334*	0.150	0.743	0.170*			0.115	0.253
**Charlson Comorbidity**		2.653*	2.622	2.683	2.593*	2.563	2.622	1.459*			1.444	1.474
**Discharge Status (vs Alive)**												
	Death	4.426*	4.362	4.491	5.854*	5.746	5.964	4.620*			4.504	4.740
**Primary Diagnosis (vs Disease-free)**												
	Respiratory	1.412*	1.394	1.431	1.633*	1.608	1.658	30.103*			29.729	30.482
	Circulatory	4.526*	4.453	4.600	4.737*	4.661	4.814	5.200*			5.120	5.282
	Neoplasms	16.134*	15.602	16.683	19.105*	18.510	19.720	16.002*			15.637	16.375
	Immunity	0.633*	0.612	0.654	0.700	0.675	0.726	6.984*			6.845	7.126
	Injury	3.480*	3.427	3.534	3.254*	3.202	3.307	2.952*			2.900	3.006
	Muscular	13.422*	13.118	13.733	12.498*	12.206	12.797	5.560*			5.403	5.722
**Number of Visit per year**		19.303*	19.180	19.426	14.133*	14.045	14.221	7.582*			7.546	7.620
**Constant Term**		0.001	0.001	0.001	0.001	0.001	0.001	0.001			0.001	0.001

*HCUs* High-Cost Users (the 96th -100th percentile), *ACUs* Average-Cost Users (the 51st -95th percentile), *LCUs* Low-Cost Users (the 1st -50th percentile)

^a^capacity: more than 500 beds

^b^capacity: 200-500 beds

^c^capacity: 10-150 beds

In terms of the characteristics of ACUs, during the prepandemic period (2016–2019), ACUs were more likely to be older, male, and admitted to hospitals outside the MoPH and health care centers. Similarly, during the COVID-19 period in 2021, ACUs also sought treatment in hospitals outside the MoPH and health care centers but, compared to LCUs, were admitted to other types of health care centers in 2021 more often than to central hospitals. Similar to HCUs, ACUs had a greater risk of mortality or greater number of comorbidities and a higher incidence of circulatory diseases, neoplasms, injuries, or muscular-related diseases as their primary diagnosis. Compared with LCUs, ACUs also had a greater number of visits per year and a greater likelihood of mortality at the end of hospitalization. Furthermore, the regions of ACUs were unstable over the study period.

**Fig. 4:**
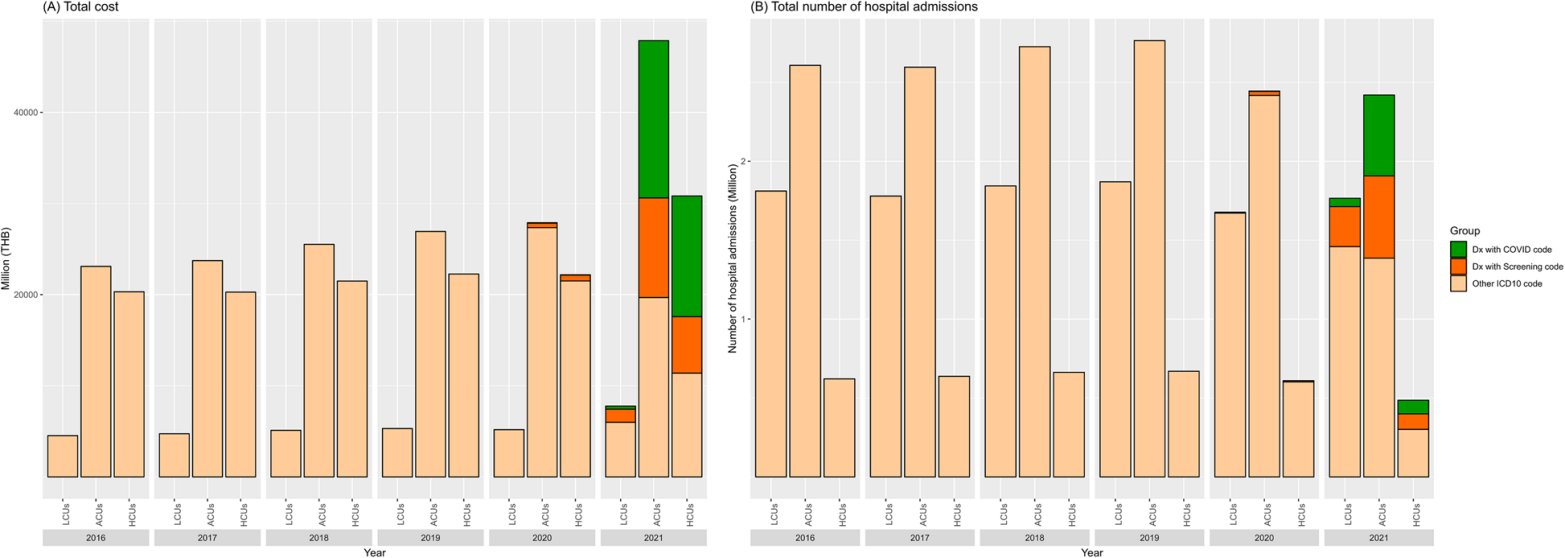
COVID-19 and healthcare cost in hospitalized patients: (**A**) total cost and (**B**) total number of hospital admissions. *Notes*: HCUs: High-Cost Users (the 96th -100th percentile); ACUs: Average-Cost Users (the 51st -95th percentile); LCUs: Low-Cost Users (the 1st -50th percentile); average rate in 2021: 31.99 THB per USD

In 2021, COVID-19 screening along with COVID-19 treatments amounted to approximately 49 billion THB, especially for LCUs (~ 2 billion THB), ACUs (~ 28 billion THB) and HCUs (~ 19 billion THB) (Fig. 4A). Additionally, ACUs had the highest number of COVID-19-related admissions due to screening in 2021. Moreover, the pandemic reduced the number of hospital admissions in 2020 and 2021 across all groups (Fig. 4B), but the total health care costs among hospitalized patients in all groups were proportionately the same.

## Discussion

When the first wave of the COVID-19 pandemic occurred in 2020, there were differences in total hospital expenditures, which increased compared with those in previous years, whereas the overall total number of hospitalizations decreased. This observation could be a cause for concern, as despite the nationwide implementation of nonpharmaceutical interventions and the limited number of nonessential hospital visits and admissions in Thailand, similar to the U.S., COVID-19 pandemic-induced surgical restrictions on operating performance were reported. However, there was still an increase in hospitalization costs in 2020.

This study confirmed that the HCUs phenomenon still existed during the pandemic in Thailand by exploring hospital expenditures from 2016 to 2021. HCUs utilized most health care resources over time [1]. Additionally, all groups of inpatients, including LCUs (the 1st-50th percentile), ACUs (the 51st-95th percentile) and HCUs (the 96th-100th percentile), continued to access health services during the pandemic, which could ensure the accessibility of health care services; however, there was a reduction in hospitalization, while the total health care costs did not decline. Although Thailand's inflation rate between 2016 and 2021 remained relatively stable due to stagnant productivity and political instability [18], the characteristics of health care utilization (HCUs) in the study were consistent with those reported in other studies, except for a difference in sex distribution compared with the Commonwealth Fund report [19]. Despite a decline in hospitalizations due to lockdown measures, the overall cost of hospitalization remained comparable. In 2021, the second year of the pandemic, which was marked by several peaks in the number of COVID-19 cases, HCUs accounted for a lower proportion of health expenditures than ACUs. Additionally, hospitals implemented stricter policies to prevent unnecessary treatments and visits [8]. This led to the prioritization of severe cases and higher admission rates for patients with respiratory diseases (J00-J99), which was associated with the quarantine period. Consequently, there was a reduction in the number of hospitalizations, including those related to HCUs. The top five primary diagnoses in HCUs from 2016–2020 were the same but differed in rank order, and circulatory-related diseases were the most common diagnoses. However, in 2021, respiratory-related diseases were the most prevalent in all groups. Additionally, the primary diagnosis as *a ‘factor influencing health status and contact with health services*’, which included health check-up services, was reported in the top five diagnoses for the first time in six years. This could be the result of the COVID-19 situation, which caused people to be admitted and undergo examinations. However, it could be difficult to distinguish COVID-19-related admissions, and comorbidities were also the underlying factor that led patients to be admitted. These findings revealed that the keys potential predictors of being an HCU were relatively the same from 2016–2021; HCUs were male, older, had a greater number of comorbidities, had a greater annual number of admissions, and had a greater number of admissions to hospitals in the central region than in Bangkok and metropolitan areas. These findings confirm that prolonged hospital stays could be a predictor of becoming an HCU, similar to those reported in studies in Canada [20, 21].

This study has several strengths, including the use of population-based study design in developing countries located in regions different from those in previous reports in developed countries. Second, this study is the first to provide important information on potential factors associated with being an HCU, ACU and LCU, in Thailand. However, this study also has several limitations that need to be considered. First, the present analysis focused only on hospitalizations because of data availability, and it may not represent overall health service utilization, which comprises both inpatient and outpatient services. Exploring outpatient visits and the medications used could be helpful in future research. Moreover, this study did not include the private sector and other public health insurance schemes, including social security schemes and civil servant benefit schemes. Thus, future research could explore this area further.

## Conclusion

This study highlights the patterns of hospitalization among HCUs, ACUs and LCUs before and during the pandemic in Thailand. The HCUs phenomenon still existed even with limited health care accessibility and lockdown measures implemented during the COVID-19 pandemic. This finding could indicate the unique need for health care services for HCUs, which differ from those of other groups. In addition, further study could help stakeholders determine the risk of being an HCU. This information can provide the foundation for future research to gain insight into how various types of procedures are being provided and whether those procedures are preventable, especially during times of crisis and when resources are scarce.

## Supplementary Information

Supplementary Material 1.

## Data Availability

No datasets were generated or analysed during the current study.

## Abbreviations

ACU: Average-cost user
COVID-19: Corona Virus 19
HCU: High-cost user
ICD-10: The International Classification of Diseases -10
LCU: Low-cost user
MoPH: Ministry of Public Health
N: Number
NHSO: National Health Security Office
SD: Standard Deviation
THB: Thai Baht
UCS: Universal Coverage Scheme
USA: The United States of America
US$: US Dollar

## References

[1] Wammes JJG, van der Wees PJ, Tanke MAC, Westert GP, Jeurissen PPT. Systematic review of high-cost patients’ characteristics and healthcare utilisation. BMJ Open. 2018;8(9):e023113. 30196269 10.1136/bmjopen-2018-023113 PMC6129088

[2] Wodchis WP, Austin PC, Henry DA. A 3-year study of high-cost users of health care. CMAJ. 2016;188(3):182–8. 26755672 10.1503/cmaj.150064 PMC4754179

[3] Ng SHX, Rahman N, Ang IYH, Sridharan S, Ramachandran S, Wang DD, et al. Characterising and predicting persistent high-cost utilisers in healthcare: a retrospective cohort study in Singapore. BMJ Open. 2020;10(1):e031622. 31911514 10.1136/bmjopen-2019-031622 PMC6955475

[4] Fan Q, Wang J, Nicholas S, Maitland E. High-cost users: drivers of inpatient healthcare expenditure concentration in urban China. BMC Health Serv Res. 2022;22(1):1348. 36376840 10.1186/s12913-022-08775-9 PMC9664649

[5] Rattanavipapong W, Wang Y, Butchon R, Kittiratchakool N, Thammatacharee J, Teerawattananon Y, et al. Retrospective secondary data analysis to identify high-cost users in inpatient department of hospitals in Thailand, a middle-income country with universal healthcare coverage. BMJ Open. 2021;11(7):e047330. 34321299 10.1136/bmjopen-2020-047330 PMC8319992

[6] Moynihan R, Sanders S, Michaleff ZA, Scott AM, Clark J, To EJ, et al. Impact of COVID-19 pandemic on utilisation of healthcare services: a systematic review. BMJ Open. 2021;11(3):e045343. 33727273 10.1136/bmjopen-2020-045343 PMC7969768

[7] Snoeijer BT, Burger M, Sun S, Dobson RJB, Folarin AA. Measuring the effect of Non-Pharmaceutical Interventions (NPIs) on mobility during the COVID-19 pandemic using global mobility data. NPJ Digit Med. 2021;4(1):81. 33986465 10.1038/s41746-021-00451-2 PMC8119480

[8] Service; DoM. Guidelines to Reduce Hospital Overcrowding to Mitigate the Spread of COVID-19. In: Service DoM, editor. Ministry of Public Health2020.

[9] Torres-Rueda S, Sweeney S, Bozzani F, Naylor NR, Baker T, Pearson C, et al. Stark choices: exploring health sector costs of policy responses to COVID-19 in low-income and middle-income countries. BMJ Glob Health. 2021;6(12):e005759. 34857521 10.1136/bmjgh-2021-005759 PMC8640196

[10] Qureshi D, Isenberg S, Tanuseputro P, Moineddin R, Quinn K, Meaney C, et al. Describing the characteristics and healthcare use of high-cost acute care users at the end of life: a pan-Canadian population-based study. BMC Health Serv Res. 2020;20(1):997. 33129316 10.1186/s12913-020-05837-8 PMC7603700

[11] Kim YJ, Park H. Improving Prediction of High-Cost Health Care Users with Medical Check-Up Data. Big Data. 2019;7(3):163–75. 31246499 10.1089/big.2018.0096

[12] Guilcher SJ, Bronskill SE, Guan J, Wodchis WP. Who Are the High-Cost Users? A Method for Person-Centred Attribution of Health Care Spending. PLoS ONE. 2016;11(3):e0149179. 26937955 10.1371/journal.pone.0149179 PMC4777563

[13] National Health Security Office. Philosophy & background. Available from: http://eng.nhso.go.th/view/1/Philosophy_Background/EN-US.

[14] Rosella LC, Fitzpatrick T, Wodchis WP, Calzavara A, Manson H, Goel V. High-cost health care users in Ontario, Canada: demographic, socio-economic, and health status characteristics. BMC Health Serv Res. 2014;31(14):532. 10.1186/s12913-014-0532-2 PMC4221677 25359294

[15] Rais S, Nazerian A, Ardal S, Chechulin Y, Bains N, Malikov K. High-cost users of Ontario’s healthcare services. Healthc Policy. 2013;9(1):44–51. 23968673 PMC3999548

[16] Tanke MAC, Feyman Y, Bernal-Delgado E, Deeny SR, Imanaka Y, Jeurissen P, Lange L, Pimperl A, Sasaki N, Schull M, Wammes JJG, Wodchis WP, Meyer GS. A challenge to all A primer on inter-country differences of high-need, high-cost patients. PLoS One. 2019;14(6):e0217353. 31216286 10.1371/journal.pone.0217353 PMC6583982

[17] StataCorp. *Stata Statistical Software: Release 16*. Version 16. College Station, TX: StataCorp LP; 2023.

[18] R Core Team. *R: A language and environment for statistical computing.* Version 1.0.7. R Foundation for Statistical Computing. Vienna: R Core Team; 2020.

[19] World Bank Group. Thailand economic monitor the road to recovery. World Bank, Bangkok. 2021. Available from: https://documents1.worldbank.org/curated/en/260291626180534793/pdf/Thailand-Economic-Monitor-The-Road-to-Recovery.pdf.

[20] Hayes SL, Salzberg CA, McCarthy D, Radley DC, Abrams MK, et al. High-Need, High-Cost Patients: Who Are They and How Do They Use Health Care?. The Commonwealth Fund. August 2016. Available from: https://www.commonwealthfund.org/publications/issue-briefs/2016/aug/high-need-high-cost-patients-who-are-they-and-how-do-they-use. 27571599

[21] Muratov S, Lee J, Holbrook A, Guertin JR, Mbuagbaw L, Paterson JM, et al. Incremental healthcare utilisation and costs among new senior high-cost users in Ontario, Canada: a retrospective matched cohort study. BMJ Open. 2019;9(10):e028637. 31662356 10.1136/bmjopen-2018-028637 PMC6830474

