# Supplementary material for: High-cost users still came to hospitals during the COVID-19 pandemic during first wave data in Thailand: secondary data analysis
Source: BMC Public Health. 2024 Oct 22;24:2917. doi: 10.1186/s12889-024-20325-y (PMC11494780; doi:10.1186/s12889-024-20325-y)
Supplement: Supplementary file 1 — Supplementary Material 1. [file 12889_2024_20325_MOESM1_ESM.docx]

**Supplementary 1:** Number of records (i.e., admissions at the hospital) for year of admission from 2006 to 2021 against year of discharge from 2016 to 2021, and the total annual expenditure in THB

| **Year of Admission​** | **Year of Discharge** | | | | | | **Total** |
| --- | --- | --- | --- | --- | --- | --- | --- |
|  | 2016​ | 2017​ | 2018​ | 2019​ | 2020​ | 2021 |  |
| 2006 to 2015​ | 55,736 | 47 | 6 | 4 | 0 | 0 | 55,793 |
| 2016​ | 4,984,128 | 55,088 | 37 | 2 | 0 | 0 | 5,039,255 |
| 2017​ | 0 | 4,958,159 | 57,216 | 42 | 6 | 1 | 5,015,424 |
| 2018​ | 0 | 0 | 5,174,903 | 56,342 | 31 | 4 | 5,231,280 |
| 2019​ | 0 | 0 | 0 | 5,246,961 | 57,436 | 29 | 5,304,426 |
| 2020​ | 0 | 0 | 0 | 0 | 4,673,957 | 56,943 | 4,730,900 |
| 2021 | 0 | 0 | 0 | 0 | 0 | 4,613,598 | 4,613,598 |
| **No. of records** | 5,039,864 | 5,013,294 | 5,232,162 | 5,303,351 | 4,731,430 | 4,670,575 | 29,990,676 |
| **N​o. of people (N)** | 3,450,630 | 3,389,480 | 3,512,581 | 3,559,710 | 3,169,905 | 3,262,705 | 20,345,011 |
| **Total expenditure (THB)** | 47,946,111,447 | 48,739,074,248 | 52,112,860,017 | 54,501,913,177 | 55,272,465,274 | 86,473,358,010 | 47,946,111,447 |

**Supplementary 2:** Flowchart of High-Cost Users (HCUs), Average-Cost Users (ACUs), and Low-Cost Users (LCUs) classification

IPD e-claim data from 2016 to 2021 (years of discharge)

No. of records = 29,990,676

No. of people = 20,345,011

Create cohorts based on years of discharge

Calculate total cost of each patient in each cohort, to carry forward costs from previous year(s) (in the case of patients who were admitted for more than a year)

Identify HCUs, ACUs, and LCUs

**Supplementary 3: Top primary diagnoses with ICD-10 during 2016-2021 among LCUs, ACUs, and HCUs**

| **Year** | **LCUs** | | | **ACUs** | | | **HCUs** | | |
| --- | --- | --- | --- | --- | --- | --- | --- | --- | --- |
|  | **ICD-10** | **Diagnosis** | **%** | **ICD-10** | **Diagnosis** | **%** | **ICD-10** | **Diagnosis** | **%** |
| **2016** | A00-B99 | Certain infectious and parasitic diseases | 18.09 | I00-I99 | Diseases of the circulatory system | 8.52 | C00-D49 | Neoplasms | 16.21 |
|  | J00-J99 | Diseases of the respiratory system | 14.63 | J00-J99 | Diseases of the respiratory system | 11.88 | I00-I99 | Diseases of the circulatory system | 22.21 |
|  | O00-O9A | Pregnancy, childbirth and the puerperium | 14.24 | K00-K95 | Diseases of the digestive system | 12.39 | J00-J99 | Diseases of the respiratory system | 11.31 |
|  | S00-T88 | Injury, poisoning and certain other consequences of external causes | 10.2 | O00-O9A | Pregnancy, childbirth and the puerperium | 8.75 | M00-M99 | Diseases of the musculoskeletal system and connective tissue | 9.63 |
|  | Z00-Z99 | Factors influencing health status and contact with health services | 8.25 | S00-T88 | Injury, poisoning and certain other consequences of external causes | 8.76 | S00-T88 | Injury, poisoning and certain other consequences of external causes | 10.03 |
| **2017** | A00-B99 | Certain infectious and parasitic diseases | 15.71 | I00-I99 | Diseases of the circulatory system | 9.25 | C00-D49 | Neoplasms | 17.05 |
|  | J00-J99 | Diseases of the respiratory system | 14.3 | J00-J99 | Diseases of the respiratory system | 12.41 | I00-I99 | Diseases of the circulatory system | 23.21 |
|  | O00-O9A | Pregnancy, childbirth and the puerperium | 10.33 | K00-K95 | Diseases of the digestive system | 12.95 | J00-J99 | Diseases of the respiratory system | 10.72 |
|  | S00-T88 | Injury, poisoning and certain other consequences of external causes | 9.04 | O00-O9A | Pregnancy, childbirth and the puerperium | 8.72 | M00-M99 | Diseases of the musculoskeletal system and connective tissue | 9.15 |
|  | Z00-Z99 | Factors influencing health status and contact with health services | 14.56 | S00-T88 | Injury, poisoning and certain other consequences of external causes | 9.2 | S00-T88 | Injury, poisoning and certain other consequences of external causes | 9.2 |
| **2018** | A00-B99 | Certain infectious and parasitic diseases | 19.72 | I00-I99 | Diseases of the circulatory system | 9.38 | C00-D49 | Neoplasms | 16.14 |
|  | J00-J99 | Diseases of the respiratory system | 14.42 | J00-J99 | Diseases of the respiratory system | 12.97 | I00-I99 | Diseases of the circulatory system | 23.5 |
|  | O00-O9A | Pregnancy, childbirth and the puerperium | 9.19 | K00-K95 | Diseases of the digestive system | 12.77 | J00-J99 | Diseases of the respiratory system | 10.67 |
|  | S00-T88 | Injury, poisoning and certain other consequences of external causes | 8.75 | O00-O9A | Pregnancy, childbirth and the puerperium | 8.1 | M00-M99 | Diseases of the musculoskeletal system and connective tissue | 10.02 |
|  | Z00-Z99 | Factors influencing health status and contact with health services | 13.02 | S00-T88 | Injury, poisoning and certain other consequences of external causes | 9.15 | S00-T88 | Injury, poisoning and certain other consequences of external causes | 9.72 |
| **2019** | A00-B99 | Certain infectious and parasitic diseases | 20.85 | I00-I99 | Diseases of the circulatory system | 9.52 | C00-D49 | Neoplasms | 16.14 |
|  | J00-J99 | Diseases of the respiratory system | 14.21 | J00-J99 | Diseases of the respiratory system | 11.77 | I00-I99 | Diseases of the circulatory system | 24.19 |
|  | O00-O9A | Pregnancy, childbirth and the puerperium | 8.42 | K00-K95 | Diseases of the digestive system | 12.66 | J00-J99 | Diseases of the respiratory system | 9.8 |
|  | S00-T88 | Injury, poisoning and certain other consequences of external causes | 8.78 | N00-N99 | Diseases of the genitourinary system | 8 | M00-M99 | Diseases of the musculoskeletal system and connective tissue | 10.32 |
|  | Z00-Z99 | Factors influencing health status and contact with health services | 12.16 | S00-T88 | Injury, poisoning and certain other consequences of external causes | 9.35 | S00-T88 | Injury, poisoning and certain other consequences of external causes | 9.56 |
| **2020** | A00-B99 | Certain infectious and parasitic diseases | 17.23 | I00-I99 | Diseases of the circulatory system | 10.65 | C00-D49 | Neoplasms | 18.43 |
|  | J00-J99 | Diseases of the respiratory system | 10.32 | J00-J99 | Diseases of the respiratory system | 9.75 | I00-I99 | Diseases of the circulatory system | 25.94 |
|  | O00-O9A | Pregnancy, childbirth and the puerperium | 9.94 | K00-K95 | Diseases of the digestive system | 13.06 | J00-J99 | Diseases of the respiratory system | 8.44 |
|  | S00-T88 | Injury, poisoning and certain other consequences of external causes | 9.87 | N00-N99 | Diseases of the genitourinary system | 8.16 | M00-M99 | Diseases of the musculoskeletal system and connective tissue | 9.92 |
|  | Z00-Z99 | Factors influencing health status and contact with health services | 12.93 | S00-T88 | Injury, poisoning and certain other consequences of external causes | 10.03 | S00-T88 | Injury, poisoning and certain other consequences of external causes | 8.5 |
| **2021** | A00-B99 | Certain infectious and parasitic diseases | 10.28 | I00-I99 | Diseases of the circulatory system | 8.13 | C00-D49 | Neoplasms | 11.37 |
|  | J00-J99 | Diseases of the respiratory system | 8.72 | J00-J99 | Diseases of the respiratory system | 23.93 | I00-I99 | Diseases of the circulatory system | 18.02 |
|  | O00-O9A | Pregnancy, childbirth and the puerperium | 9.87 | K00-K95 | Diseases of the digestive system | 9.01 | J00-J99 | Diseases of the respiratory system | 40.43 |
|  | S00-T88 | Injury, poisoning and certain other consequences of external causes | 10.77 | S00-T88 | Injury, poisoning and certain other consequences of external causes | 7.43 | S00-T88 | Injury, poisoning and certain other consequences of external causes | 4.57 |
|  | Z00-Z99 | Factors influencing health status and contact with health services | 13.55 | Z00-Z99 | Factors influencing health status and contact with health services | 15.16 | Z00-Z99 | Factors influencing health status and contact with health services | 5.99 |
|  |  |  |  |  |  |  |  |  |  |
|  |  |  |  |  |  |  |  |  |  |
